# Radiological characteristics of coronavirus patients

**DOI:** 10.1097/MD.0000000000034267

**Published:** 2023-07-14

**Authors:** Gulden Bilgin, Zuhal Yavuzdagli, Ismail Arslan

**Affiliations:** a Department of Chest Diseases, Ankara Training and Research Hospital, Ankara, Turkey; b Department of Family Medicine, Ankara Training and Research Hospital, Ankara, Turkey.

**Keywords:** COVID-19, diagnosis, RT-PCR, thoracic CT

## Abstract

The present study aimed to evaluate the radiological findings of coronavirus patients who had positive computed tomography of the lung following real-time reverse transcriptase-polymerase chain reaction testing. The data of 1727 patients who had reverse-transcriptase-polymerase chain reaction (RT-PCR) testing between May 2020 and August 2021 and had thoracic computed tomography (CT) on Days 7th to 8th were analyzed retrospectively. The Radiological Society of North America’s recommended reporting system was used to categorize CT findings. Of the 1727 patients who underwent RT-PCR testing, there were 1417 patients with positive CT results. Of these 1417 patients, 679 (47.9%) were female. When patients with high blood values were evaluated, the number of CT-positive patients was significantly higher than CT-negative patients (*P* < .05). The number of patients with low lymphocyte and albumin values was significantly higher (*P* < .05). In 75.7% of those who had positive CT results, the PCR result was positive. Thoracic CT is a critical diagnostic tool in Coronavirus Disease 2019 patients with RT-PCR negative. It also depicts the progression of lung involvement in RT-PCR-positive patients. Performing it late in the disease’s progression may increase the risk of contracting the disease.

## 1. Introduction

Coronavirus Disease 2019 (COVID-19), which was first observed in Wuhan, China, is an infectious disease caused by the coronavirus SARS-CoV-2, which causes severe acute respiratory syndrome.^[1]^ The World Health Organization declared it a pandemic after it spread first in China and then globally.^[2]^ The most common symptoms are dry cough, fever, myalgia, dyspnea, and sore throat, while less common symptoms include nasal congestion, headache, runny nose, vomiting, and diarrhea.^[3]^

Mild cases do not have lung involvement. Patients usually present with symptoms of an upper respiratory tract infection. Fever may be mild or absent. In mild and moderate cases, O_2_ saturation exceeds 90%.^[4]^

In severe cases, dyspnea and/or hypoxemia usually appear 1 week after the onset of the disease, followed by septic shock and acute respiratory distress syndrome, arrhythmia, and acute cardiac damage. Tachypnea is >30/min and hypoxemia with spO_2_ <90%. Persistent fever, diffuse inflammatory response markers like dimer and ferritin, and an increase in proinflammatory cytokines have all been linked to death.^[4]^

Among laboratory findings, poor prognosis criteria include lymphopenia, elevated liver enzymes, elevated lactate dehydrogenase, elevated inflammatory markers (C reactive protein, ferritin, etc), elevated dimer, prolonged prothrombin, elevated troponin, elevated creatine phosphokinase, and acute kidney injury.^[5]^

Because all these symptoms are nonspecific and the disease is progressing quickly, diagnostic tests are required. Polymerase chain reaction (PCR) test’s sensitivity for clinical application ranges between 37% and 71%.^[6]^ Computed tomography (CT) is widely used in the diagnosis and evaluation of complications.^[7,8]^

The Radiological Society of North America (RSNA) has proposed 4 major imaging classifications for COVID-19 have been developed.^[9]^ Typical features of COVID-19 pneumonia include peripheral, bilateral, and ground-glass opacities, whereas atypical features include rarer features (isolated lobar or segmental consolidation without ground-glass opacities, small nodules, cavity, smooth interseptal thickening with pleural effusion), and nonspecific imaging modalities for COVID-19 pneumonia (multifocal diffuse, perihilar or unilateral ground-glass opacities that do not show a specific distribution pattern, are not round shaped or peripheral, and are multifocal, diffuse, perihilar or unilateral).^[3–9]^

The aim of our study is to evaluate the laboratory and thorax CT imaging findings of COVID-19 patients who had reverse-transcriptase-polymerase chain reaction (RT-PCR) testing between May 2020 and August 2021 at the University of Health Sciences Ankara Training and Research Hospital.

## 2. Materials and Methods

Our study is a retrospective one. University of Health Sciences Ankara Training and Research Hospital Ethics Committee granted permission on June 7, 2021 with project number E-21-365. The application to the Ministry of Health’s scientific research platform was also approved, with the form number 2021-03-01T07_31_20.

Between May 2020 and August 2021, data from 1727 patients who admitted to our hospital with symptoms of fatigue, dry cough, dyspnea, fever, headache, runny nose, sore throat, vomiting, and diarrhea were obtained; 1727 patients had RT-PCR testing and had thoracic CT on Days 7th to 8th were analyzed retrospectively.

All patients underwent CT examinations with a 16-detector slice Toshiba CT device at a low dose, with images taken without contrast and in the supine position. The protocol tube voltage was set to 120 kV, and the slice thickness was set to 1 mm.

When evaluating the available CT findings, the RSNA-recommended report language was used.^[10]^

Data obtained in our study were analyzed with the SPSS 21.0 package program. The Kappa statistic was used to assess the agreement between the 2 methods, and the chi-square dependency test was used to assess the relationship between the variables. *P* < .05 value indicates significant compatibility.

## 3. Results

Of the 1727 patients who underwent RT-PCR testing, there were 1417 patients with positive CT results. Of these 1417 patients, 679 (47.9%) were female. The mean age of females was 59.7 ± 15.6 years, while the mean age of males was 58.5 ± 15.0 years. Sore throat and back pain were the most common symptoms in patients with positive CT results.

When patients with high blood values were evaluated, the number of CT-positive patients was significantly higher than CT-negative patients (*P* < .05). The number of patients with low lymphocyte and albumin values was significantly higher (*P* < .05). The patients with positive laboratory values among the patients diagnosed with CT are shown in Table 1.

**Table 1:** The number of patients with positive laboratory values among the patients diagnosed with CT

n = 1417	n	%
Sedimentation	999	70.50
CRP	1122	79.18
LDH	1014	71.55
Leukocyte	441	31.12
Lymphocyte*	606	42.76
AST	486	34.30
ALT	494	34.86
Urea	402	28.37
Creatinine	322	22.72
D-dimer	863	60.90
Fibrinogen	623	43.96
Ferritin	523	36.90
Na	606	42.76
Albumin*	486	34.30

CRP = C reactive protein, CT = computed tomography, LDH = lactate dehydrogenase.

* Low results were considered positive.

In CT-positive patients, the following characteristics were more common: fibrous appearance, nodule, cobblestone, vascular appearance, pleural effusion, air bronchogram, reverse halo finding, ground glass, consolidation, an association of ground glass and consolidation, and unilateral and bilateral involvement (*P* < .05).

When the morphologic features of the lesions on CT were examined, the most common typical finding (81.90%) was ground glass. Cobblestone was the most common atypical finding (25.93%). Table 2 shows the lesions discovered on CT.

**Table 2:** Morphologic features of lesions on CT

	n	%
Typical (n = 1116)		
Ground-glass	914	81.90
Ground-glass and consolidation	589	52.78
Consolidation	648	58.06
Vascular enlargement	459	41.12
Atypical (n = 54)		
Lobar or segmental consolidation	4	7.40
Nodule	11	20.37
Fibrosis	7	12.96
Pleural effusion	5	9.26
Reverse Halo	3	5.56
Cobblestone	14	25.93
Air bronchogram	10	18.52
Uncertain (n = 247)		

CT = computed tomography.

According to the regions of involvement on CT, there was 78.79% bilateral and 21.24% unilateral lung involvement. There was 97.74% peripheral involvement.

In total, 88% of the left lung and 86% of the right lung were affected.

Lesions were found in 64% of the upper zone, 82% of the middle zone, and 86% of the lower zone.

CT results were positive in 82.05% of those with positive RT-PCR results. In 75.7% of those who had positive CT results, the PCR result was positive.

According to the RSNA, the results of the 310 patients included in the study were normal. Table 3 shows the classification according to the RSNA.

**Table 3:** COVID-19 imaging category according to RSNA

n = 1727	n	%
Typical appearance	1116	64.62
Atypical appearance	54	3.13
Uncertain appearance	247	14.30
Normal	310	17.95

RSNA = Radiological Society of North America.

## 4. Discussion

After the first 4 days of upper respiratory tract infection, the RT-PCR method may occasionally fail due to errors in virus detection in lower respiratory tract samples and in sampling technique.^[4–9,11]^ Our study shows that using laboratory and radiologic findings facilitates the diagnostic process.

Ground-glass appearance, organized pneumonia pattern, and nodular or mass-like ground-glass areas located bilaterally and peripherally were the most common radiological findings of COVID-19 in thoracic CT. Furthermore, involvements such as cobblestone appearance and reverse halo finding may be observed.^[11]^

CT sensitivity was found to be 96% in the study conducted by Li et al and 98% in the study of Fang et al.^[8,9,11,12]^ CT sensitivity was 82% in our study. The reason for the low sensitivity percentage in our study may be the late application of the patients.

Typical findings are those that have been described as more common and specific in COVID-19 pneumonia. Bilateral, peripheral, and multifocal (patchy) foci in the middle and lower zones, ground-glass areas, consolidation, a combination of both findings, and vascular enlargement are the most common.^[10,13]^ Most of the patients’ CT reports in our study were reported as typical findings.

Atypical findings are those that are uncommon or nonexistent in COVID-19 pneumonia but are more common in other diseases. Lobar or segmental consolidation, cavitation, and tree-in-bud appearance with centrilobular nodules are examples of such findings.^[10,13]^ The number of patients with atypical findings in our study was extremely low.

The absence of any parenchymal findings related to infection on thorax CT indicates a negative status for pneumonia. In the early stages of COVID-19, no CT findings may be present.^[9]^

The prevalence and types of chest CT findings differ depending on the stage of infection at the time of imaging. Most patients do not have CT findings within the first 2 days of symptom onset.^[3–9,11]^

Pleural effusion and mediastinal lymphadenopathy are uncommon conditions. In our study, we found no evidence of mediastinal lymphadenopathy. Complications should generally be considered in the presence of pleural effusion.^[14]^ Pleural effusion was found in 9.26% of CT-diagnosed cases.

Since the disease’s CT imaging features might be comparable to those of other causes of acute lung injury and organizing pneumonia, it may be difficult to interpret.^[14,15]^ Some viral pneumonia, particularly acute lung injury patterns with influenza and organizing pneumonia, are the main differential diagnoses.^[14]^

Ground-glass opacity in the form of peripherally and posteriorly located nodules or masses is typical of COVID-19 CT findings. Ground-glass opacities are frequently circular in shape. It is possible to see a cobblestone appearance with thickened interlobular and interlobar septal thickening. Cobblestone appearance is observed in 5% to 36% of cases. When seen with diffuse ground-glass and consolidation, it indicates disease progression or peak period.^[10,14,15]^

Ground-glass opacity was found in 88% of patients in a meta-analysis by Salehi et al, and was reported as the most common imaging finding^[16]^: Similarly, the most common imaging finding in our study was ground-glass opacity.

The rate of ground glass was 83.1%, the association of ground glass and consolidation was 50.72%, and the rate of consolidation was 56.84% in thoracic CT in our study, whereas in a different study by Li, ground glass was found in 35% of cases, the association of ground glass area and consolidation was 55%, and the rate of consolidation was 6%.^[12]^ In a Chinese study, the ground glass rate was 46% and the consolidation rate was 50% in 888 patients. The ground glass rate was 88% and the consolidation rate was 32% in the study by Salehi et al^[16]^ Ground-glass area was 99% in an Italian study, consolidation was 83%, and the association of ground-glass field and consolidation was 79%.^[10]^

Vascular enlargement describes the enlargement of peripheral vascular structures close to or within the ground-glass area, as well as consolidation. Some studies use a diameter of more than 3 mm.^[17]^ Vascular enlargement was found to be 3.5% in a study by Heng Meng et al,^[18]^ 82% in a study by Li et al,^[12]^ and 37.24% in our study.

Air bronchograms found in COVID-19 patients are bronchial structures secondary to mucus plugs that become partially dilated and more visible, especially in the consolidated lung parenchyma. They were discovered in between 21% and 80% of the studies.^[19]^ The rate in our study was 18.18%.

For COVID-19 patients, cobblestone in the ground-glass area and consolidation may indicate progression.^[15]^ In studies, this finding was found to be 5% to 36%. This rate was found to be 31.82% in our study.

The term “reverse halo appearance” refers to a ring-like ground-glass area or consolidation and is seen in other pneumonia. In studies, this rate ranged between 2% and 3%.^[15]^ The rate of reverse halo in our study was 4.54%. This could be due to the addition of secondary infections as a result of the late CT scan.

A nodule is an opacity <3 cm in diameter with a smooth or irregular border in the lung parenchyma. This is a common finding in viral pneumonia. According to research, it ranges between 3% and 13%.^[15]^ The rate of nodules in our study was found to be 20.45%.

Some studies have reported pleural effusion and mediastinal lymphadenopathy as poor progression findings.^[10,13]^ Pleural effusion was 9.09% in our study, and no patients had mediastinal lymphadenopathy .

## 5. Conclusion

In conclusion, while thorax CT is not recommended for screening purposes as an imaging modality, it is frequently used in practice in conjunction with clinical and laboratory findings for diagnosis and evaluation of complications. In addition to RT-PCR in the early stage, CT in the late period (7–8 days after the onset of the disease) plays a significant role.

## Author contributions

**Conceptualization:** Gulden Bilgin, Zuhal Yavuzdagli, Ismail Arslan.

**Data curation:** Gulden Bilgin, Zuhal Yavuzdagli, Ismail Arslan.

**Formal analysis:** Gulden Bilgin, Zuhal Yavuzdagli, Ismail Arslan.

**Funding acquisition:** Gulden Bilgin, Zuhal Yavuzdagli, Ismail Arslan.

**Investigation:** Gulden Bilgin, Zuhal Yavuzdagli.

**Methodology:** Gulden Bilgin, Zuhal Yavuzdagli, Ismail Arslan.

**Resources:** Zuhal Yavuzdagli.

**Software:** Ismail Arslan.

**Supervision:** Gulden Bilgin.

**Validation:** Gulden Bilgin, Zuhal Yavuzdagli, Ismail Arslan.

**Visualization:** Ismail Arslan.

**Writing – original draft:** Gulden Bilgin, Zuhal Yavuzdagli, Ismail Arslan.

**Writing – review & editing:** Gulden Bilgin, Ismail Arslan.
